# Economic crisis and obesity in the Canary Islands: an exploratory study through the relationship between body mass index and educational level

**DOI:** 10.1186/s12889-019-8098-x

**Published:** 2019-12-30

**Authors:** Aránzazu Hernández-Yumar, Ignacio Abásolo Alessón, Beatriz González López-Valcárcel

**Affiliations:** 1 Departamento de Economía Aplicada y Métodos Cuantitativos, Facultad de Economía, Empresa y Turismo, Universidad de La Laguna (ULL), San Cristóbal de La Laguna, Santa Cruz de Tenerife, Spain; 2 Departamento de Métodos Cuantitativos en Economía y Gestión, Universidad de Las Palmas de Gran Canaria (ULPGC), Las Palmas de Gran Canaria, Spain

**Keywords:** Obesity, Body mass index, Economic crisis, Social gradient, The Canary Islands

## Abstract

**Background:**

The Canary Islands is one of the Spanish Regions with the highest obesity prevalence, and one of the Autonomous Communities that was hit hard by the economic crisis that arrived to Spain in 2008. This research studies the education-related inequalities in adult obesity in the Canary Islands and their evolution in recent years, considering the possible impact of the economic recession.

**Methods:**

A repeated cross-sectional analysis is carried out with data obtained from the Canary Islands Health Surveys of 2004, 2009 and 2015. Obesity is measured through the body mass index (BMI). The analysis is performed using linear regression models for the general population and by gender, adjusting by age, educational attainment and island of residence. Likewise, the models also include dummy variables for each year and the corresponding interactions between the years and the education variable.

**Results:**

The results show a decrease in the obesity prevalence in 2015 compared to 2009 (from 19.54 to 18.64%). An increase in the BMI of the population and that of women (+ 0.33 and + 0.59 units, respectively) in 2009, as well as a decline in the BMI of women with medium education (− 0.21 units) are also observed. Besides, there is an inverse correlation between education and BMI, and statistically significant differences among some islands.

**Conclusions:**

Obesity figures in the Canary Islands have decreased and women have been more greatly affected by the changes in BMI during the economic crisis. Due to the fact that educational attainment is a protective factor in general (and for women with medium education levels in times of crisis, in particular), regional authorities should implement actions that promote access to education and healthy lifestyles, paying attention to territorial disparities.

## Background

Obesity has largely increased over the last years in Spain [1]. In addition, obesity has not only been a national concern, but it has also become a severe regional health problem, as a result of the high obesity prevalence rates in some Spanish regions, such as the Canary Islands, Andalusia or Extremadura [2–4]. The situation in the Canary Islands is particularly worrying. Although the Nutritional Study of the Spanish Population (ENPE) [5] shows a reduction up to 20,1% in the obesity prevalence in these Islands between 2014 and 2015, this region was one of the three autonomous communities with the highest percentage of obese people in Spain during the first decade of the twenty-first century [2–4]. In fact, results from DARIOS (Dyslipidemia, Atherosclerosis Risk, elevated high-sensitivity C-reactive protein, and Inflammatory and Oxidative Status in the Spanish population) study reveal that 32% of men and 36% of women were categorised as obese in the Canary Islands between 2000 and 2005 [2]. In addition, this region also presents higher mortality rates by chronic diseases such as diabetes mellitus type 2, cancer and cardiovascular diseases [6], which are closely related to obesity.

The analyses of obesity cannot be detached from the individual’s socioeconomic and demographic characteristics, but neither from the socioeconomic situation within the geographic territory to which they belong. Firstly, individual characteristics play a relevant role in the prevalence of obesity. Many studies have established the existence of a socioeconomic gradient in Spain. That is, an inverse relationship between socioeconomic characteristics of individuals, such as income or educational achievement, and their body mass index (BMI) [7–16], especially accentuated by education [8, 10, 13, 14, 16] and more predominant in women [7, 8, 10, 15–17]. Particularly, in the Canary Islands, Darias-Curvo [18] establishes that, in 2004, while university education and income are protective factors against obesity for women, education and income seem to have no effect on obesity among men.

Secondly, the economic context of the crisis, in which the Spanish population has been involved since 2008, has negatively affected the living conditions of people [19, 20]. The Canary Islands was one of the regions that most suffered from the impact of this recession. In addition, the arrival of the economic crisis in Spain conditioned, to a certain extent, the health status of individuals [21–23]. The studies that have analysed the effects of economic recessions on obesity prevalence have generated some controversy, as there seems to be no agreement on this. Although some studies suggest that economic crises cause increases in obesity rates [11, 21, 24, 25], others indicate that these crises generate the opposite effect: decreases in obesity [26, 27]. Among the explanatory reasons of the increase in obesity figures during economic crisis are changes in eating habits. The decline in household income and the economic impoverishment of families can cause a deterioration of the diet of people, generating situations of malnutrition, due to excessive energy intake or insufficient amount of nutrients ingested, which facilitate the appearance of obesity [21, 28].

We have developed this research because of the scarcity of studies on obesity in adults that consider the possible effects of the economic crisis in the Canary Islands. Therefore, our main aim is to expand the knowledge about obesity among adults in this region and its evolution in recent years, especially throughout the economic recession, taking into account the relationship between obesity and educational attainment.

## Methods

### Population

This study was carried out with data from the Canary Islands Health Surveys of 2004 [29], 2009 [30] and 2015 [31], developed by the Canary Islands Institute of Statistics (ISTAC) and the Canary Islands Health Service. Data were taken between June and August of 2004 [32], between October 2009 and January 2010 [33], and between October 2014 and March 2015 [32]. These surveys collect information, through personal interviews, of adults and children, who were randomly selected among residents of the Canary Islands. After dropping individuals under 18 and the missing values in the variables under study, we constructed a data pool with a sample of 3995 (2004), 4468 (2009) and 4507 (2015) adults. From an economic perspective, we can divide the studied period into pre-crisis (2004), crisis (2009) and post-crisis (2015).

### Variables

The Word Health Organization (WHO) proposes the body mass index (BMI) as the most useful obesity indicator, because it can be applied among adults regardless of age and gender [34]. A BMI equal to or over 25 indicates that the individual is overweight, while if it is equal to or greater than 30, the individual is obese. Therefore, the BMI is our continuous dependent variable, which is calculated dividing the self-reported weight (kg) by the self-reported height (m^2^). In addition, we use other variables in the analysis such as gender, age, educational attainment and the island of residence. Although family income is an economic variable often used in this kind of analysis, we have excluded it because the Canary Islands Health Survey of 2004 does not provide information about income.

As for gender, it is categorised as male and female, and age is a continuous variable that goes from 18 onwards. The participants were also classified in the following three groups according to their educational attainment: (i) low education, which includes primary education or less; (ii) medium education, which comprises compulsory secondary education or equivalent, upper secondary education and professional education or equivalent; and (iii) high education, which contains any level of university education. Besides, the island of residence variable refers to each of the seven Canary Islands: El Hierro, La Gomera, La Palma, Tenerife, Gran Canaria, Lanzarote and Fuerteventura.

Finally, we have created dummy variables for the years 2009 and 2015 and for the interactions between educational achievements and these years.

### Statistical analysis

We conducted a repeated cross-sectional analysis by performing a linear regression model for the whole population and for each gender, using the Stata 15 statistical software. In order to study the evolution of obesity in the entire sample during recent years, considering 2004 as the year of reference, we have built this model as follows:

$$ {y}_i={\beta}_0+{\beta}_1{women}_i+{\beta}_2{age}_i+{\beta}_3 ag{e^2}_i+{\beta}_4{ME}_i+{\beta}_5{HE}_i+{\beta}_6{2009}_i+{\beta}_7{2015}_i+{\beta}_8 El\_{Hierro}_i+{\beta}_9 La\_{Gomera}_i+{\beta}_{10} La\_{Palma}_i+{\beta}_{11} Gran\_{Canaria}_i+{\beta}_{12}{Lanzarote}_i+{\beta}_{13}{Fuerteventura}_i+{\beta}_{14}{ME}_i\ast {2009}_i+{\beta}_{15}{HE}_i\ast {2009}_i+{\beta}_{16}{ME}_i\ast {2015}_i+{\beta}_{17}{HE}_i\ast {2015}_i+{e}_i $$

where *y*_*i*_ is the BMI of the individual *i* (*i* = 1, . . ., N) and *β*_0_ is the intercept. In addition, the model also contains a dummy variable for gender. We have included age and age-squared to allow for the non-linear relationship between age and the body mass index. Besides, we have added the educational attainment as fixed effects, *ME* referring to medium education and *HE* to high education. Based on previous studies, the omitted educational category is low education (LE) [12–14]. A dummy variable for each Island has also been included, taking Tenerife as reference, as this island has the largest number of individuals of the sample. Finally, we have also incorporated dummies for years 2009 and 2015, and the interactions of educational attainment with each year to assess the statistical significance of changes in the effect of education on BMI over time.

In addition, because previous studies have demonstrated the existence of differences in BMI between men and women [1–5], we built one model for males and another for females to analyse the BMI by gender.

## Results

**Fig. 1:**
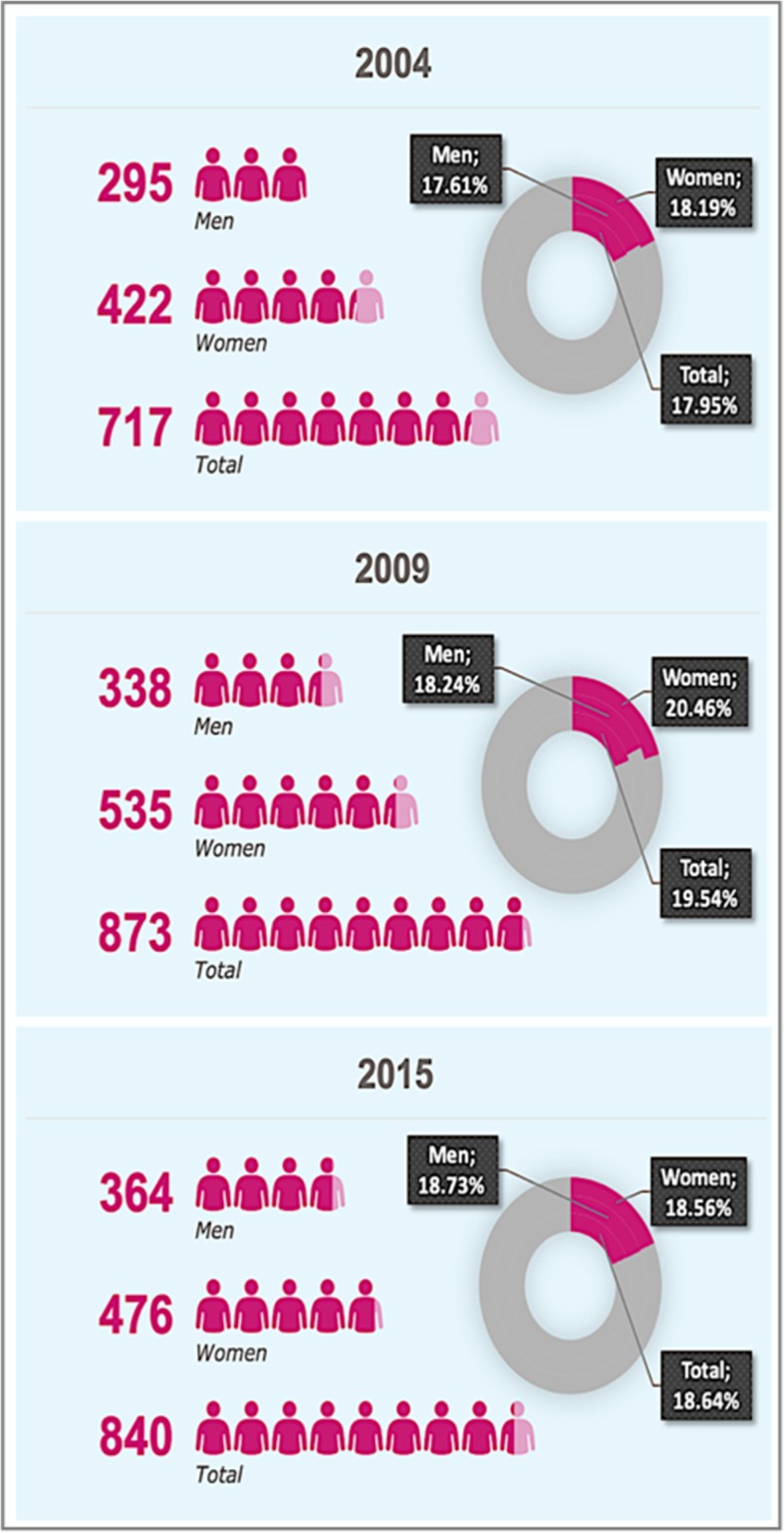
Obese people in the Canary Islands in 2004, 2009 and 2015. Pink area shows the proportion of people who are obese, while grey area is related to non-obese people. The percentages refer to the total number of people of their respective group. Source: Prepared by the authors with data from the Canary Islands Health Surveys of 2004, 2009 and 2015

This analysis on BMI carried out for the region of the Canary Islands shows that the total percentage of obese people increased from 2004 to 2009 in almost 2 percentage points (i.e., from 17.95 to 19.54%), but decreased to 18.64% in 2015, still being a higher prevalence than in 2004 (Fig. 1). This particular trend can be observed among women, although with larger variations, but not among men, whose prevalence of obesity rose since 2004 to 2015 monotonically.

We have studied a sample of the Canary Islands population, where 40% are men and 60% women, approximately (Table 1). Besides, men have an average age of 46–50 years and women of 48–52 years.

**Table 1:** Individual characteristics and mean body mass index (BMI) by sex

	Total					
	2004		2009		2015	
	n (%)	mean BMI (95% CI)	n (%)	mean BMI (95% CI)	n (%)	mean BMI (95% CI)
Total	3995	26.06 (25.91–26.2)	4468	26.32 (26.18–26.46)	4507	26.27 (26.13–26.4)
Age (mean, years)	47.5		49.2		51.6	
Educational attainment						
Low education	1569 (39.27)	27.22 (26.97–27.46)	1648 (36.88)	27.71 (27.47–27.96)	1384 (30.71)	27.53 (27.27–27.79)
Medium Education	1944 (48.66)	25.56 (25.35–25.76)	2157 (48.28)	25.66 (25.47–25.86)	2347 (52.07)	25.98 (25.80–26.17)
High education	482 (12.07)	24.30 (23.95–24.65)	663 (14.84)	24.97 (24.67–25.27)	776 (17.22)	24.88 (24.61–25.16)
Island of residence						
El Hierro	211 (5.28)	25.93 (25.34–26.52)	195 (4.36)	27.37 (26.78–27.96)	220 (4.88)	26.28 (25.71–26.85)
La Gomera	231 (5.78)	25.85 (25.32–26.39)	198 (4.43)	26.13 (25.44–26.81)	213 (4.73)	26.36 (25.82–26.89)
La Palma	333 (8.34)	27.05 (26.50–27.59)	275 (6.15)	26.25 (25.65–26.85)	282 (6.26)	26.03 (25.49–26.57)
Tenerife	1374 (34.39)	25.80 (25.54–26.05)	1628 (36.44)	26.07 (25.84–26.3)	1566 (34.75)	25.96 (25.74–26.19)
Gran Canaria	1273 (31.86)	26.37 (26.10–26.64)	1544 (34.56)	26.58 (26.34–26.83)	1564 (34.70)	26.57 (26.34–26.81)
Lanzarote	311 (7.78)	25.38 (24.91–25.84)	336 (7.52)	26.07 (25.55–26.59)	328 (7.28)	26.35 (25.87–26.83)
Fuerteventura	262 (6.56)	25.72 (25.20–26.24)	292 (6.54)	26.05 (25.53–26.57)	334 (7.41)	26.34 (25.81–26.87)
Men						
Total	1675 (41.93)	26.42 (26.22–26.62)	1853 (41.47)	26.53 (26.34–26.72)	1943 (43.11)	26.63 (26.44–26.81)
Age (mean, years)	46.6		47.8		50.6	
Educational attainment						
Low education	617 (36.84)	27.17 (26.82–27.51)	609 (32.87)	27.13 (26.78–27.48)	538 (27.69)	27.5 (27.12–27.88)
Medium Education	861 (51.40)	25.99 (25.72–26.27)	946 (51.05)	26.24 (25.99–26.50)	1093 (56.25)	26.4 (26.16–26.65)
High education	197 (11.76)	25.92 (25.40–26.44)	298 (16.08)	26.22 (25.82–26.62)	312 (16.06)	25.9 (25.49–26.31)
Island of residence						
El Hierro	72 (4.30)	26.61 (25.58–27.63)	82 (4.43)	27.43 (26.71–28.16)	95 (4.89)	27.10 (26.25–27.94)
La Gomera	95 (5.67)	26.57 (25.83–27.30)	83 (4.48)	26.10 (25.15–27.04)	81 (4.17)	26.26 (25.54–26.98)
La Palma	106 (6.33)	27.41 (26.59–28.23)	119 (6.42)	26.00 (25.25–26.76)	129 (6.64)	26.22 (25.51–26.93)
Tenerife	558 (33.31)	26.19 (25.84–26.54)	624 (33.68)	26.27 (25.95–26.58)	679 (34.95)	26.41 (26.08–26.73)
Gran Canaria	585 (34.93)	26.52 (26.17–26.88)	634 (34.21)	26.87 (26.55–27.19)	641 (32.99)	26.96 (26.64–27.27)
Lanzarote	135 (8.06)	26.10 (25.45–26.76)	161 (8.69)	26.80 (26.13–27.47)	154 (7.93)	26.38 (25.73–27.03)
Fuerteventura	124 (7.40)	26.2 (25.56–26.85)	150 (8.09)	26.09 (25.44–26.73)	164 (8.44)	26.71 (26.04–27.38)
Women						
Total	2320 (58.07)	25.80 (25.59–26.01)	2615 (58.53)	26.16 (25.96–26.36)	2564 (56.89)	26.00 (25.81–26.19)
Age (mean, years)	48.1		50.1		52.3	
Educational attainment						
Low education	952 (41.03)	27.25 (26.92–27.59)	1039 (39.73)	28.06 (27.73–28.38)	846 (33)	27.55 (27.21–27.9)
Medium Education	1083 (46.68)	25.21 (24.91–25.5)	1211 (46.31)	25.21 (24.93–25.49)	1254 (48.91)	25.62 (25.35–25.88)
High education	285 (12.28)	23.18 (22.75–23.61)	365 (13.96)	23.95 (23.56–24.35)	464 (18.1)	24.20 (23.85–24.56)
Island of residence						
El Hierro	139 (5.99)	25.58 (24.86–26.30)	113 (4.32)	27.32 (26.44–28.20)	125 (4.88)	25.66 (24.89–26.43)
La Gomera	136 (5.86)	25.36 (24.62–26.10)	115 (4.40)	26.15 (25.17–27.12)	132 (5.15)	26.42 (25.68–27.15)
La Palma	227 (9.78)	26.88 (26.18–27.58)	156 (5.97)	26.44 (25.55–27.33)	153 (5.97)	25.88 (25.08–26.67)
Tenerife	816 (35.17)	25.53 (25.17–25.89)	1004 (38.39)	25.95 (25.64–26.26)	887 (34.59)	25.62 (25.31–25.93)
Gran Canaria	688 (29.66)	26.24 (25.84–26.64)	910 (34.80)	26.38 (26.03–26.74)	923 (36.00)	26.31 (25.97–26.65)
Lanzarote	176 (7.59)	24.82 (24.19–25.45)	175 (6.69)	25.40 (24.64–26.17)	174 (6.79)	26.32 (25.62–27.03)
Fuerteventura	138 (5.95)	25.29 (24.49–26.08)	142 (5.43)	26.01 (25.18–26.84)	170 (6.63)	25.99 (25.18–26.79)

Regarding education, data show an overall increase in educational attainment among women and men over the studied period. Medium education and, especially, high education have increased in the Canary Islands (i.e., + 7% and + 43%, respectively) (Table 1). In the case of women, there was an increment in high education of 47% in 2015 compared to 2004, with the largest increase since 2009. Men show different behaviour, because the increase in the education among males occurred between 2004 and 2009 (i.e., + 37% of men with high education).

Data for the mean BMI of Table 1, which ranges from 25.8 to 26.6, points to a problem of overweight among the Canary population. Besides, by educational attainment, the mean population BMI decreases as the education level increases, which is also observed in men and women. This indicator of obesity experienced an increase from 2004 to 2015 and a decrease of lesser magnitude since 2009, both in the entire population and in women. As for the BMI of men, it increased until 2015. In addition, despite women presenting higher rates of obesity (except in 2015), men have the highest average BMI in all years.

The results of the linear regression models (Table 2) show a statistically significant difference in BMI between men and women. The BMI of women is 0.13 units lower than that of men. Besides, a quadratic relationship between BMI and age is observed. The mean BMI increases with age, until a certain year, when the BMI reaches its maximum value and begins to decrease, although in small magnitude (i.e., − 0.002 BMI units per year).

**Table 2:** β coefficients and 95% Confidence Intervals of the linear regression models

	Total		Men		Women	
	β	95% CI	β	95% CI	β	95% CI
Intercept	20.58^a^	19.87–21.28	19.91^a^	18.96–20.85	20.19^a^	19.21–21.17
Gender	− 0.13^a^	− 0.16 - −0.10	–	–	–	–
Age	0.23^a^	0.21–0.26	0.25^a^	0.22–0.29	0.22^a^	0.18–0.26
Age squared	− 0.002^a^	− 0.002 - −0.0015	− 0.002^a^	− 0.0024 - −0.0017	− 0.002^a^	− 0.002 - −0.0013
Educational attainment						
LE	Ref.		Ref.		Ref.	
ME	−0.80^a^	−1.12 - −0.48	− 0.49^b^	− 0.93 - −0.05	− 1.06^a^	− 1.50 - −0.61
HE	−2.22^a^	−2.69 - −1.75	− 0.85^b^	− 1.51 - −0.20	− 3.18^a^	−3.83 - −2.52
Island of residence						
El Hierro	0	− 0.38 - 0.37	0.50^c^	− 0.03 - 1.04	− 0.32	− 0.85 - 0.20
La Gomera	− 0.46^b^	− 0.83 - −0.08	− 0.35	− 0.87 - 0.18	− 0.50^c^	−1.02 - 0.02
La Palma	0.24	−0.08 - 0.57	0.07	−0.39 - 0.53	0.39^c^	−0.05 - 0.84
Tenerife	Ref.		Ref.		Ref.	
Gran Canaria	0.46^a^	0.27 – 0.64	0.41^a^	0.15–0.67	0.51^a^	0.25–0.77
Lanzarote	0.10	− 0.21 - 0.41	0.22	−0.20 - 0.63	0.04	−0.41 - 0.49
Fuerteventura	0.29^c^	−0.03 - 0.62	0.21	−0.21 - 0.63	0.40	−0.08 - 0.88
Year						
2004	Ref.		Ref.		Ref.	
2009	0.33^b^	0.02–0.64	− 0.15	−0.61 - 0.3	0.59^a^	0.16–1.01
2015	0.06	−0.27 - 0.39	0.12	−0.34 - 0.59	0	−0.45 - 0.45
LE2009	Ref.		Ref.		Ref.	
ME2009	−0.41^c^	− 0.82 - 0.01	0.25	−0.34 - 0.83	− 0.80^c^	−1.38 - −0.22
HE2009	0.12	−0.50 - 0.73	0.25	−0.60 - 1.11	0.01	−0.85 - 0.87
LE2015	Ref.		Ref.		Ref.	
ME2015	−0.14	−0.56 - 0.29	−0.17	− 0.76 - 0.42	−0.09	− 0.69 - 0.5
HE2015	0.15	−0.46 - 0.76	−0.46	−1.32 - 0.40	0.63	−0.21 - 1.47

*LE* refers to low education, *ME* to medium education and *HE* to high education, *CI* refers to confidence intervals.

^a^ Statistical significance at 99% confidence; ^b^ Statistical significance at 95% confidence; ^c^ Statistical significance at 90% confidence

Regarding changes in BMI over time, the only statistically significant variations took place between 2004 and 2009 among the whole population and for women (Table 2). The results indicate that the mean BMI of the Canary Islands increased 0.33 BMI units in 2009 compared to 2004, and 0.59 BMI units in women.

In addition, the statistically significant negative coefficients of the regression models establish a negative correlation between BMI and education (Table 2). However, differences between group averages are more accentuated among women. While men report a coefficient below 1 both in medium education (ME) and high education (HE), women with ME and HE present a reduction in their BMI of − 1.06 and − 3.18 units, respectively, as compared with those with low education (LE). In addition, when we analyse the changes in BMI over time considering educational achievement, it is found that women with medium education decreased their BMI by − 0.21 units (=0.59 − 0.80) in 2009, with respect to those with low education.

Finally, regarding the place of residence, considering Tenerife as the Island of reference, people from Gran Canaria and Fuerteventura report a higher BMI, and those from La Gomera show a lower BMI (Table 2). Particularly among men, those from El Hierro and Gran Canaria have a higher BMI than those from Tenerife (i.e. 0.50 and 0.41 BMI units more, respectively). In the case of women, while females have a higher BMI in La Palma and Gran Canaria than in Tenerife (i.e. 0.39 and 0.51 BMI units more, respectively), women from La Gomera report a lower BMI (i.e., 0.50 BMI units less) than in the island of reference.

## Discussion

The percentage of people who are obese has increased throughout the last decades around the world uninterrupted since the 80s, and Spain has not been an exception [1, 35]. However, our analysis indicates that the trend in prevalence of obesity followed by the Spanish Region of the Canary Islands differs from that of the national level [1]. Despite the continuous increase in obesity in Spain, our results confirm the decrease in the obesity figures in these Islands, in the last few years, shown in the ENPE study [5].

Likewise, our study show differences in obesity by gender also in this particular region: obesity was more prevalent in women in 2004 and 2009, which is in line with the results of the DARIOS Study [2] for the Canary population, between 2000 and 2005, while men from the Canary Islands report the highest obesity prevalence in 2015, as in Spain [1, 5], although with a slight difference with respect to women. Regarding BMI, the results point to the fact that women present a lower mean BMI than men, which coincides with the results for Spain [4, 5] but differs to those of the DARIOS study [2] for the population of the Canary Islands. However, it should be borne in mind that this latter research only considers the period between 2000 and 2005.

We can observe that an inverse relationship between education and BMI is established in our analysis. The results indicate that the BMI decreases as educational level increases, in both men and women, which contrasts to Darias Curvo’s study [18]. However, that analysis only used data from 2004. Despite this correlation existing in both genders, having higher studies is considered a stronger protective factor against obesity in women. This can be perceived by comparing the coefficients of men and women: while the BMI of males with HE is 0.85 units lower compared to that of males with LE, the BMI of females with university studies is 3.18 units lower than that of females with LE. We can therefore corroborate that a greater effect of educational attainment on BMI among women, shown elsewhere [7, 8, 10, 15–17], is also observed in the Canary Islands. There is an unknowledge of the harmful impact of unhealthy behaviour on people’s health in many cases, and this lack of information is more predominant in social groups with low education [17], which may be associated to this gradient by education levels. The higher the educational attainment of an individual, the greater their critical ability to make healthy decisions, their self-perception of their health and their understanding of the risks of obesity [17, 36–38]. Thus, education has a great influence on obesity and plays a protective role against it.

The results also indicate that the BMI of the people of the Canary Islands increases between 2004 and 2009. In addition to the individual characteristics, the economic and social context surrounding individuals can also exert some influence on the population BMI. An economic crisis arrived to Spain in 2008. Some of its visible consequences in these Islands were sharp increases in the unemployment rates (i.e., + 149% in 2009 with respect to 2007) [39], decrements in the remuneration of employees [40] and in the available gross household income [41], and decreases in the Gross Domestic Product (GDP) [42] between 2009 and 2012. Although the impact of the crisis seemed to be greater since 2009, the economy of the islands had already shown signs of deceleration from a year earlier [39–42]. These effects of this economic crisis, among other, were detrimental for the living conditions of the population, affecting the socioeconomic status of individuals and generating a quality loss in the diet of population and, consequently, an increment in the BMI. The deterioration of the diet as a result of the household income loss (in many case derived from becoming unemployed [28]) is recognised in previous studies [21, 43]. This together with the limited access to information, described above, can explain the increase in BMI in 2009.

Despite the general rise of the women’s BMI in 2009, we can observe that women with medium education were more protected than those with low education during the first years of the crisis. This could have been caused by the larger impact of the economic crisis on the most disadvantaged groups [19, 20].

On the other hand, the slight recovery of the economy since 2013, as well as the different preventive regulations that authorities and other institutions have implemented in recent years [44–49], could have resulted in the decrement in the obesity prevalence observed in the results for 2015. These preventive actions have been designed to tackle obesity by means of promoting a healthy lifestyle, where the unbalanced diets and sedentary lifestyle have no presence. These two factors greatly condition the individual BMI, as weight is mainly the result of the difference between the intake and the expenditure of calories.

Finally, there are statistically significant differences between some islands, which means that the territory of residence has a significant influence on BMI. Future research should focus on the assessment of socioeconomic and demographic characteristics of each island, to improve the understanding of the differences in BMI between them.

This study has some limitations. The BMI results may be underestimated, due to the self-reported bias, because people tend to misreport their height and weight [50–53]. In addition, we have not been able to include the individual’s income in the analysis. However, it can also be said that, first, the results about the BMI of the Canary Islands Health Surveys follow the same trend as those of the ENPE study [5], which uses non self-reported data and considers a period after 2010. As mentioned above, the BMI in the Canary Islands decreases in recent years in both analyses. And secondly, due to the high correlation between education and income, the effects of education can also include some influence of income. In addition, among these individual socioeconomic characteristics, educational attainment affects the BMI of a person to a greater extent [8, 10, 13, 14, 16].

Another limitation is that we used independently cross-sectional data, therefore we cannot control for unobserved individual heterogeneity. Some omitted individual characteristics that might influence obesity could bias the estimates of the included explanatory variables -age, education, island.

In addition, we must bear in mind that in this sample women are overrepresented. The actual distribution of the population of the Canary Islands by gender in these years was 50% women and not 60%, as in our sample.

## Conclusions

The prevalence of obesity in the Canary Islands has not followed the same trend as in Spain. In these Islands, obesity rates increase in 2009 (crisis period) and decrease in 2015, that is, in the post-crisis period. Despite this reduction, obesity rates remain worryingly high: around 19% of the population has a BMI equal to or greater than 30. Nevertheless, the mean BMI of the Canary Islands mainly reflects a problem of overweight among their population.

By gender, women show a lower mean BMI than men and have been largely affected by the changes in BMI. The BMI of women showed an increase during the first years of the economic crisis (except that of women with medium education) but decreased in line with an improvement in the economy of the Islands. Men did not experience any significant variation.

Finally, a social gradient by educational attainment has been established in the Canary Islands. Education is a protective factor against obesity, especially in women. In addition, those women with medium education were more protected than those with low education in time of crisis. Considering the great influence of education on the BMI, regional authorities should facilitate and promote access to education and healthy lifestyles, trying to eliminate the barriers that limit the access to information on the benefits of a balanced diet and physical activity and the costs of not adopting such recommendations, thus raising the awareness of the health problems caused by obesity. Besides, public policies to mitigate the economic effects of crises on obesity are also of great relevance, at least, to avoid increases in the obesity prevalence. Further, it is essential that the policy makers integrate an insular perspective in the design of any intervention focused on tackling obesity.

## Data Availability

The data analysed in this study were obtained from the Canary Islands Health Surveys of 2004, 2009 and 2015, and are publicly available upon request to the Canary Islands Institute of Statistics (ISTAC) (http://www.gobiernodecanarias.org/istac/temas_estadisticos/sociedad/salud/estadodesalud/C00035A.html).

## Abbreviations

BMI: Body mass index
CI: Confidence interval
DARIOS: Dyslipidemia, Atherosclerosis Risk, elevated high-sensitivity C-reactive protein, and Inflammatory and Oxidative Status in the Spanish population
ENPE: Nutritional Study of the Spanish Population
GDP: Gross domestic product
HE: High education
ISTAC: The Canary Islands Institute of Statistics
LE: Low education
ME: Medium education
WHO: World Health Organization
